# A Comparison of Techniques for Collecting Skin Microbiome Samples: Swabbing Versus Tape-Stripping

**DOI:** 10.3389/fmicb.2018.02362

**Published:** 2018-10-02

**Authors:** Kazuhiro Ogai, Satoshi Nagase, Kanae Mukai, Terumi Iuchi, Yumiko Mori, Miki Matsue, Kayo Sugitani, Junko Sugama, Shigefumi Okamoto

**Affiliations:** ^1^Wellness Promotion Science Center, Institute of Medical, Pharmaceutical and Health Sciences, Kanazawa University, Kanazawa, Japan; ^2^Department of Clinical Laboratory Science, Faculty of Health Sciences, Institute of Medical, Pharmaceutical and Health Sciences, Kanazawa University, Kanazawa, Japan; ^3^Department of Clinical Nursing, Faculty of Health Sciences, Institute of Medical, Pharmaceutical and Health Sciences, Kanazawa University, Kanazawa, Japan; ^4^Advanced Health Care Science Research Unit, Innovative Integrated Bio-Research Core, Institute for Frontier Science Initiative, Kanazawa University, Kanazawa, Japan

**Keywords:** skin microbiome, swabbing, tape stripping, bacterial culture, next generation sequencing

## Abstract

The swabbing and tape-stripping methods have traditionally been used for collecting skin microbiome samples for skin bacterial analysis, although no reports have compared the outcome of these methods for collecting skin bacteria. Our purpose was to show the differences in microbial composition between samples collected using the swabbing and tape-stripping methods, by both the next generation sequencing and culture studies. The skin microbiome was collected by both methods, and the samples were processed for a sequence-based microbiome analysis and culture study. The next-generation sequencing results showed that skin bacteria collected using the tape-stripping method were comparable to those collected using the swabbing method. In the culture study, the tape-stripping method collected a greater number and wider variety of viable skin bacteria than the swabbing method. These results suggest that the tape-stripping method is comparable to the swabbing method for collecting viable skin bacteria, without losing fidelity to the composition of skin microbiome.

## Introduction

Skin serves as a body “shield” preventing the evaporation of body fluids and protecting our body from external insults (Segre, 2006). The barrier function of the skin is not limited in its physical hardness; the commensal bacteria on the skin also play an important role for skin barrier and immunological reactions (Grice and Segre, 2011). Commensal bacteria on the skin, such as *Staphylococcus epidermidis*, *Propionibacterium acnes*, and *Corynebacterium* spp., are reported to prevent the colonization of pathogenic bacteria (Sanford and Gallo, 2013), retain water in the stratum corneum (Scheimann et al., 1960), regulate skin pH (Nodake et al., 2015), and contribute to the immune response (Belkaid and Segre, 2014). In contrast, the imbalance between commensal and pathogenic bacteria (i.e., dysbiosis) can cause several skin disorders (Schommer and Gallo, 2013). In the context of such interactions between skin bacteria and diseases, understanding the nature of skin bacteria is now considered crucial to maintaining skin health.

Analysis of the skin microbiome was originally based on the culture method, in which the bacteria on the skin was collected by a swab and plated onto the appropriate media. With recent advances in DNA-sequencing techniques such as next-generation sequencing (NGS), it is now possible to know the whole population of skin microbiome (Grice et al., 2008; Costello et al., 2009; Capone et al., 2011; The Human Microbiome Project Consortium, 2012). However, the bacterial culture method is still widely used as one of the clinical laboratory tests, because viable bacteria are indispensable for an antibiotic-susceptibility test. Cultured bacteria are also required in a virulence test and genetic and proteomic analyses. Therefore, both culture methods and exhaustive microbiome analysis are considered essential for studies on bacteria (Lagier et al., 2015).

Collection of the skin microbiome samples is commonly performed using the swabbing method (Gao et al., 2007; Grice et al., 2008; Van Horn et al., 2008; Costello et al., 2009; Capone et al., 2011; The Human Microbiome Project Consortium, 2012; Fitz-Gibbon et al., 2013; Lagier et al., 2015). Although the relative proportions of skin bacterial species reflected using the swabbing method are comparable to those reflected using a skin biopsy, the yield of viable bacteria using the swabbing method is reportedly lower than that using other methods such as the pad scrubbing and cylinder suspension methods (Whyte et al., 1989; Hambraeus et al., 1990). Another problem of the swabbing method is that the condition of swabbing (i.e., pressure, direction, number of times swabbed) cannot be well controlled. The collection efficiency can be greatly affected by pressure, the way and number of strokes, and even by the swab material (Van Horn et al., 2008). Therefore, it is plausible that the swabbing method may not necessarily be the best method for collecting skin bacteria. As an alternative, the tape-stripping method has been used in several studies including culture studies (Updegraff, 1967; Lange-Asschenfeldt et al., 2011) and NGS analysis (Chng et al., 2016; Tanaka et al., 2016). This method was considered to be better in terms of uniformity and quantity for collecting skin fungi (Tajima et al., 2008); however, this may not be the case for skin bacteria because the number, position, and depth of fungi may differ from those of bacteria. Although the swabbing, biopsy, and scrape methods have been compared for skin microbiome analysis (Grice et al., 2008), there is little direct evidence illustrating the similarity of the skin microbiome, both in culture and NGS studies, between samples collected using the swabbing method and those collected using the tape-stripping method.

Therefore, in this study, we attempted to compare the swabbing and tape-stripping methods using an NGS analysis and a culture study.

## Materials and Methods

### Ethical Consideration

The whole process of this study, including the human skin microbiome analysis, was approved by the Medical Ethics Committee of Kanazawa University (approval No. 632-4). The collection of skin microorganisms was performed by a researcher of the same sex as the participant for privacy protection. This study was conducted in accordance with the Declaration of Helsinki and the Microorganism Safety Management Regulations of Kanazawa University. Bacterial samples were processed in a biosafety level-2 laboratory.

### Participants

We recruited seven healthy young participants (three men and four women; age 21–29 years) who provided written informed consent. No participants had skin disorders, such as psoriasis or atopic dermatitis, or any systemic disorders. In addition, no participants reported the use of topical or systemic antibiotics at the time of the examination.

### Collection of Skin Bacteria

#### Position

In this study, skin bacteria were collected from the back skin (**Supplementary Figure 1**) of each participant using the two different methods: the swabbing and tape-stripping methods (**Supplementary Figure 2**). All collection procedures were performed by a trained researcher.

#### Swabbing Method

Skin bacteria were collected by the swabbing method as described in previous studies (Grice et al., 2009; Capone et al., 2011) with slight modifications. In brief, a 4.4 × 4.4-cm square on the designated area (**Supplementary Figure 1**) was gently swabbed with a cotton swab soaked in 0.9% sodium chloride with 0.1% Tween-20 in a Z-stroke manner (Rushing, 2007). For the culture study, the swab head was immersed in 500 μL of a sterile saline solution for shaking out the bacteria, followed by centrifugation at 8,000 rpm for 10 min to collect the bacterial pellet. For DNA extraction, the swab head was cut off and stored in a sterile 1.5-mL centrifugation tube at −80°C until DNA extraction (Capone et al., 2011; Bassiouni et al., 2015; **Supplementary Figure 2A**).

#### Tape-Stripping Method

Collection of skin bacteria by the tape-stripping method was based on the method described previously (Updegraff, 1967; Lange-Asschenfeldt et al., 2011) with modifications. First, medical air-permeable tape with acrylic glue (4.4 × 4.4 cm) were sterilized by ultraviolet radiation. The sterility of the tape and absence of bacterial DNA were confirmed at an early stage (**Supplementary Figure 3**). Then, three sterilized tapes were applied to each designated region of the participant’s skin (**Supplementary Figure 1**) for 1 min. Two tapes were then peeled off from the skin with sterile forceps and applied to individual sheep blood agar plates [trypticase soy agar (Becton, Dickinson and Company, NJ, United States) with 5% sterile defibrinated sheep blood (Nippon Bio-Supp. Center, Tokyo, Japan)]. These media were cultured as described in the next section. The remaining one tape was peeled off and was stored in a sterile 1.5-mL centrifugation tube at −80°C until DNA extraction (**Supplementary Figure 2B**).

### Bacterial Culture

#### Culture From a Swab

The collected bacterial pellet from the swab was suspended in 200 μL of a sterile saline solution and then spread on two sheep blood agar plates. One plate was cultured at 37°C for 3 days to culture aerobic bacteria, whereas the other plate was cultured in an anaerobic jar at 37°C for 5 days to culture anaerobic bacteria. At appropriate time points, all the bacterial colonies on the medium were suspended in 5 mL of a sterile saline solution. Then, the bacterial pellet was formed using 1 mL of the bacterial suspension by centrifugation at 7,500 rpm for 10 min. The bacterial DNA was then extracted for bacterial species identification.

#### Culture From Adhesive Tape

The bacteria obtained with the tape were cultured and collected by the same manner as the swab culture. The tape was remained attached to the medium during culturing.

#### Colony Counting

The number of colonies on each medium was counted as follows. First, each medium was photographed by using a digital camera (IXY 640; Canon Inc., Tokyo, Japan) after 3 days culture for aerobic bacteria or 5 days for anaerobic bacteria. After that, the number of colonies on the image was determined by the manual cell counting function by using ImageJ software (version 1.52b) (Schneider et al., 2012).

### DNA Extraction

The whole DNA was extracted by means of a QIAamp DNA Mini Kit (QIAGEN N.V., Venlo, Netherlands) in accordance with the appendix protocol “Isolation of genomic DNA from Gram-positive bacteria.” In brief, the collected swab cotton, tape, or bacterial pellet was first treated with 180 μL of an enzyme solution [20 mg/mL lysozyme (Wako Pure Chemical Industries, Ltd., Osaka, Japan) and 200 μg/mL lysostaphin (Wako Pure Chemical Industries, Ltd.) in 20 mM Tris–HCl (pH 8.0), 2 mM EDTA, and 1.2% Triton-X 100] at 37°C for 30 min with intermittent vortexing. Next, 20 μL of Proteinase K and 200 μL of Buffer AL were added to the tube, incubated at 56°C for 30 min, followed by deactivation of the enzymes at 95°C for 15 min. The resultant solution was then processed for the DNA extraction according to the manufacturer’s instructions. The concentration of extracted DNA was quantified with a Qubit^®^ dsDNA HS Assay Kit using Qubit^®^ 3.0 (Thermo Fisher Scientific, Inc., MA, United States).

### Real-Time PCR

To determine the copy number of the 16S rRNA gene in the DNA extracted from the swab or adhesive tape, real-time PCR was performed. The 16S rRNA gene was amplified using universal primer pairs (F: 5′-ACTGAGAYACGGYCCA-3′; R: 5′-CTGCTGGCACGDAGTTAGCC-3′) (Wang and Qian, 2009) and a universal probe (5′-VIC-ACTGCTGCCTCCCGTA-NFQMGB-3′) (Gao et al., 2010) with the Thunderbird^®^ Probe qPCR Mix (Toyobo Co., Ltd., Osaka, Japan). A standard curve was drawn from a known amount of the 16S rRNA gene [100, 10, 1, and 0.1 pg of *Propionibacterium acnes* genomes, which are equivalent to 7.23 × 10^4^, 7.23 × 10^3^, 7.23 × 10^2^, and 7.23 × 10^1^ 16S rRNA genes, respectively (Nadkarni et al., 2002; Miura et al., 2010; Stoddard et al., 2015)]. All the reactions were performed with the Mx3005P System (Agilent Technologies, CA, United States). The copy number of 16S rRNA gene was compared for the same size of skin area (4.4 × 4.4-cm square; **Supplementary Figure 1**, open squares).

### Identification of Bacterial Species From Cultured Colonies

Species of cultured bacteria were determined by species-specific PCR identification. In brief, 1 μL of extracted DNA, species-specific primer sets (0.2 μM each; **Supplementary Table 1**; specificity was as shown in **Supplementary Figure 4**), and SapphireAmp^®^ Fast PCR Master Mix (TaKaRa Bio Inc., Shiga, Japan) were used for amplification of the target genes using a thermal cycler (GeneAtlas G02; Astec Co., Ltd., Fukuoka, Japan). The amplification conditions were: 94°C for 1 min followed by 25 cycles of (98°C for 10 s, 68°C for 10 s) for *Bacillus subtilis* primer set; 94°C for 1 min followed by 25 cycles of (98°C for 5 s, 60°C for 5 s, and 72 °C for 10 s) for the other primer sets. The amplified products were electrophoresed on 2% agarose gel and visualized with the GeneGenius 2 Bio Imaging System (Syngene, MD, United States).

### NGS for 16S rRNA Gene

The extracted DNA samples from the swab and adhesive tape were processed for 16S rRNA gene sequencing. In brief, the hypervariable region 3 to 4 (V3–V4; approximately 460 bp) of the 16S rRNA gene (Castelino et al., 2017) was first amplified with *Ex Taq*^®^ Hot Start Version (TaKaRa Bio Inc.) and the 1st PCR primers [F: 5′-ACACTCTTTCCCTACACGACGCTCTTCCGATCT-**CCTACG GGNGGCWGCAG**-3′; R: 5′-GTGACTGGAGTTCAGACG TGTGCTCTTCCGATCT-**GACTACHVGGGTATCTAAKCC**-3′ consisted of the Illumina paired-end adapter sequences (underlined) and 16S rRNA gene-specific sequences (bold)] using a thermal cycler (GeneAtlas G02). The 1st PCR mixture consisted of: 10.0 μL of template, 5.0 μL of 10 × reaction buffer, 4.0 μL of 10 mM dNTPs, 1.0 μL of 10 μM primers (each), 0.25 μL of 5 U/μL *Ex Taq* enzyme, and 28.75 μL of nuclease- and DNA-free water. The thermal condition of the 1st PCR was as follows: 94°C for 2 min; 25 cycles of (94°C for 30 s, 50°C for 30 s, and 72°C for 60 s); 72°C for 5 min. The amplified fragments were purified with a NucleoSpin^®^ Gel and PCR Clean-up kit (MACHEREY-NAGEL GmbH & Co. KG, Düren, Germany) and dedicated to the 2nd PCR. The 2nd PCR was performed with the purified 1st PCR solution as a template and barcoded primer sets (**Supplementary Table 2**). The 2nd PCR mixture was as the same formula in the 1st PCR. The thermal condition of the 2nd PCR was as follows: 94°C for 2 min; 10 cycles of (94°C for 30 s, 59°C for 30 s, and 72°C for 60 s); 72°C for 5 min. After purification and quantification of the DNA concentration with a Qubit^®^ dsDNA HS Assay Kit using Qubit^®^ 3.0 (Thermo Fisher Scientific, Inc.), the equimolar mixture of all PCR products was sent to an outsourcing laboratory (FASMAC Co., Ltd., Kanagawa, Japan) for Illumina MiSeq 16S amplicon sequencing. All raw sequences were deposited in DNA Data Bank of Japan (DDBJ; accession number is DRA006958).

### Microbiome Analysis

#### Sequence Filtering and Chimera Elimination

The raw pair-end sequences were filtered (Q score > 20) using sickle (version 1.3) (Joshi and Fass, 2011) and combined using PANDAseq (version 2.11) (Masella et al., 2012). Next, the chimeric sequences were eliminated using USEARCH (version 8.0.1623_i86linux64) (Edgar, 2010) with the chimera-checked operational taxonomic units (OTUs) database of Greengenes (version 13.8, 97_otus.fasta) (DeSantis et al., 2006). The non-chimeric sequences were finally filtered by their size (>300 bp accepted) followed by the analysis with Qiime (version 1.9.1) (Caporaso et al., 2010).

#### 16S rRNA Gene Amplicon Analysis

The nonchimeric sequences were first clustered into OTUs with a 97% similarity using the “pick_de_novo_otus.py” command. The representative sequences of each OTU were picked followed by the assignment of taxonomy with the Greengenes OTU database (97_otus.fasta). The global singletons (i.e., OTUs that appeared only one time in the whole OTU Table) were excluded from the OTU Table. The relative and absolute abundance matrices of each sample were constructed with the “summarize_taxa.py” command. For the alpha diversity analysis, the samples were rarefied at 9,840 depth (minimum read number among all samples) followed by a calculation of the number of observed OTUs, phylogenetic diversity (PD_whole_tree), Chao1 index (Chao, 1984), and Shannon diversity index. For the beta diversity analysis, the weighted UniFrac distance matrix (Lozupone et al., 2007) was calculated followed by visualization by the principal coordinate analysis (PCoA).

### Statistics

Data are shown as the means ± standard deviations [or the 25th, 50th, 75th percentile boxes with 25th percentile - 1.5 × interquartile range (IQR) to 75th percentile + 1.5 × IQR whisker in the box plot] unless otherwise stated. The beta diversity plot was prepared by using Origin Pro software (version 2018b; OriginLab Corp., MA, United States). The number of colonies collected using the swabbing and tape-stripping methods was compared by using the Wilcoxon signed-rank test with the R statistical package (version 3.4.1) (R Core Team, 2016). The concentration of DNA and copy number of the 16S rRNA gene were compared between the two methods by using the Wilcoxon signed-rank test using R. The (dis)similarity of the bacterial composition between the two methods was assessed by the correlation analysis of the bacterial relative abundance using the “compare_taxa_summaries.py” command implemented in Qiime (10,000 simulations), Yue and Clayton theta index (*𝜃*_YC_) (Yue and Clayton, 2005), and permutational multivariate analysis of variance (perMANOVA) (“adonis” command in “vegan” package (The Comprehensive R Archive Network, 2016); 10,000 simulations for a *P*-value calculation) using R (Hoppe et al., 2015). The relative abundance of each taxon by each method was compared by using the paired Wald test in “DESeq2” package (Love et al., 2014), followed by the adjustment of *P*-values by Benjamini–Hochberg’s false discovery rate control (Benjamini and Hochberg, 1995; Bassiouni et al., 2015), denoted herein as *q*-values. The indices of alpha diversity were compared by a paired *t*-test using R. The Mantel test was used to compare the distance matrices [“mantel” command in “vegan” package; Pearson’s correlation coefficient (*r*), 10,000 simulations for *P*-value calculation]. A *P*-value < 0.05 or *q*-value < 0.05 was considered statistically significant.

## Results

### NGS studies

#### The Yield of DNA and Copy Number of the 16S rRNA Gene

**FIGURE 1:**
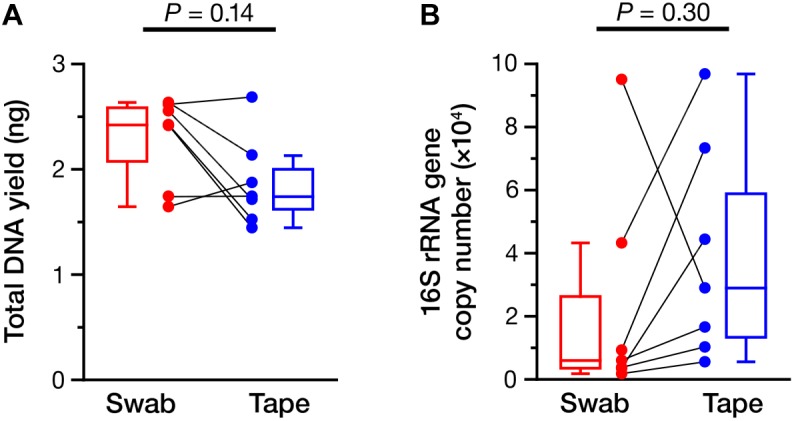
Yield of total DNA and 16S rRNA gene. **(A)** The amount of total DNA collected using the swabbing and tape-stripping methods. **(B)** The copy number of the 16S rRNA gene obtained by the swabbing and tape-stripping methods. The data points of the swabbing and tape-stripping methods from the same participant are connected.

We sought to perform a detailed NGS analysis on the samples obtained using the swabbing and tape-stripping methods to confirm whether the bacterial composition was different between the two methods. Prior to NGS, the yield of total DNA and copy number of the 16S rRNA gene were determined (**Figure 1**) and were found to be equivalent between the swabbing and tape-stripping methods (total DNA: **Figure 1A**, *P* = 0.14; 16S rRNA gene: **Figure 1B**, *P* = 0.30). There was no significant correlation in the yield of DNA between the two methods (**Supplementary Figure 5A**; Spearman’s rho = 0.39, *P* = 0.38). However, we could find a significant correlation in the number of 16S rRNA gene if we exclude one sample (**Supplementary Figure 5B**; Spearman’s rho = 0.83, *P* = 0.042, *n* = 6).

#### Bacterial Composition

**FIGURE 2:**
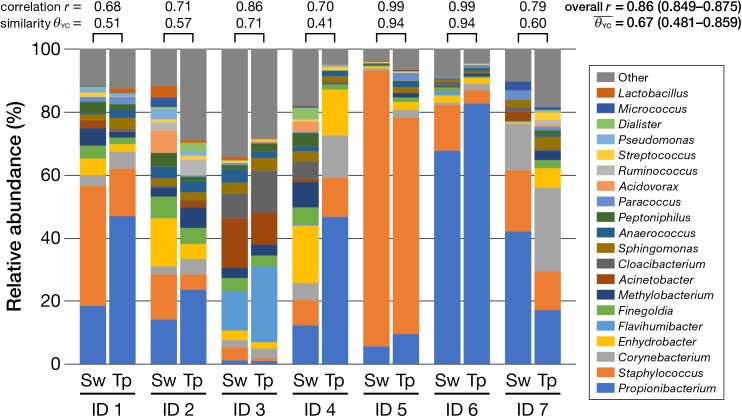
Relative abundance of bacteria classified at the genus level (top 20). Note the high similarity between the swabbing and tape-stripping methods within each participant, indicated by the Yue and Clayton theta index (*𝜃*_YC_) and Pearson’s correlation coefficient (*r*). Sw, swabbing method; Tp, tape-stripping method.

The obtained 16S rRNA gene amplicons were analyzed by the MiSeq sequencing [cluster density: 815 ± 35 K/mm^2^; clusters passing filter (%): 93.96 ± 0.69; Q ≥ 30 (%): 75.1; read numbers of each sample were as in **Supplementary Table 3**]. Next, the bacterial composition obtained by using a swab was compared with that obtained by using adhesive tape. **Figure 2** shows the bacterial compositions of each participant classified by the different collection methods (the swabbing and tape-stripping methods). Apparently, similar compositions were obtained using the swabbing and tape-stripping methods. The correlation of the bacterial relative abundance between the two methods was high [*r* = 0.86 with 95% confidence interval (CI) of 0.849–0.875, *P* = 0.0001]. The similarity indices [*𝜃*_YC_; ranges from 0 (dissimilar) to 1 (similar)] of the bacterial composition between the two methods were considerably high (average *𝜃*_YC_ = 0.67, 95% CI: 0.481–0.859). The perMANOVA analysis revealed no significant difference in bacterial compositions between the two methods (*P* = 0.87). In detailed analysis, the relative abundances of each observed genus (whose total relative abundance was > 0.1%) were compared; there were no significant differences in the relative abundances between the two methods (**Table 1**).

**Table 1:** Comparison of the relative abundance of selected bacteria (>0.1% in total) between the swabbing and tape-stripping methods.

Genus	Average of relative abundance (%)		*P*-value^†^	*q*-value^‡^
	Swab	Tape		
*Propionibacterium*	22.89	32.39	0.94	1.00
*Staphylococcus*	26.65	16.87	0.016^∗^	0.13
*Corynebacterium*	4.23	8.31	0.50	1.00
*Enhydrobacter*	6.46	4.91	0.59	1.00
*Flavihumibacter*	2.11	3.41	0.011^∗^	0.12
*Finegoldia*	3.08	2.36	0.79	1.00
*Methylobacterium*	2.87	2.46	0.78	1.00
*Acinetobacter*	3.24	1.83	0.20	0.82
*Cloacibacterium*	2.41	2.23	0.07	0.47
*Sphingomonas*	1.99	2.51	0.93	1.00
*Anaerococcus*	1.47	1.85	0.15	0.78
*Peptoniphilus*	1.95	1.21	0.99	1.00
*Paracoccus*	0.90	1.05	0.84	1.00
*Acidovorax*	1.49	0.19	0.59	1.00
*Bacteroides*^§^	0.50	1.03	0.005^∗∗^	0.10
*Ruminococcus*	0.40	0.94	1.00	1.00
*Streptococcus*	0.52	0.80	0.56	1.00
*Pseudomonas*	0.72	0.51	0.92	1.00
*Dialister*	0.70	0.51	0.92	1.00
*Micrococcus*	0.96	0.23	0.006^∗∗^	0.10
*Lactobacillus*	0.70	0.47	0.98	1.00
*Dermacoccus*	0.25	0.80	0.17	0.78
*Mycobacterium*	0.47	0.56	0.84	1.00
*Blautia*	0.00	0.54	0.71	1.00
*Bifidobacterium*	0.01	0.41	0.79	1.00
*Rhodococcus*	0.22	0.19	0.55	1.00
*Prevotella*	0.16	0.17	0.37	1.00
*Granulicatella*	0.25	0.05	0.51	1.00
*Halomonas*	0.21	0.08	0.26	0.96
*Luteococcus*	0.08	0.18	0.93	1.00
*Nocardioides*	0.10	0.14	1.00	1.00
*Kocuria*	0.08	0.15	0.85	1.00
*Parabacteroides*	0.02	0.19	0.79	1.00

*^∗^P < 0.05, ^∗∗^P < 0.01. ^†^Paired Wald test in “DESeq2” package of R. ^‡^Adjusted P-value by means of Benjamini–Hochberg’s false discovery rate control. ^§^ Including “genus 1–68” (“Bacteroides coagulans” according to the BLAST alignment).*

#### Beta Diversity

**FIGURE 3:**
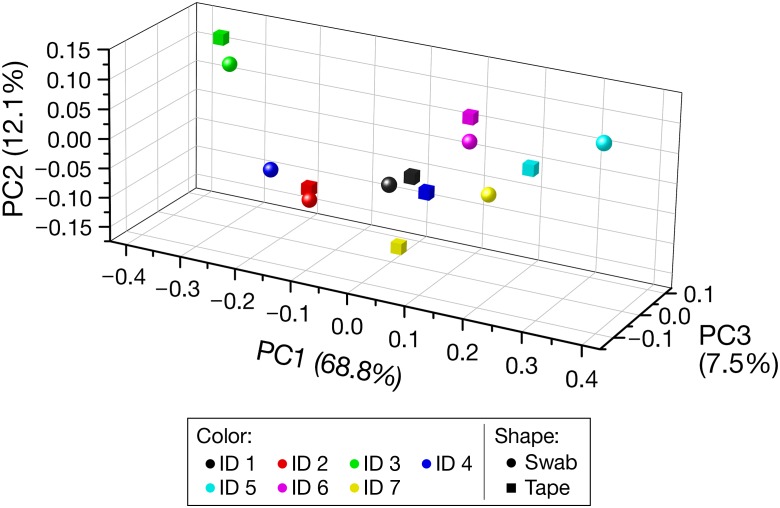
Beta diversity based on the weighted UniFrac distance. Each color denotes each participant. Spheres and cubes indicate the data obtained from the swabbing and tape-stripping methods, respectively. PC, principal coordinate.

The PCoA plot based on the weighted UniFrac distance (**Figure 3**) indicated the closeness of the PCoA points of the swabbing and tape-stripping methods in each participant. The Mantel test for the weighted UniFrac distance matrix showed moderate but significant correlation (*r* = 0.61, *P* = 0.025), which further confirmed the closeness of the distance matrices derived from the two methods.

#### Alpha Diversity

**FIGURE 4:**
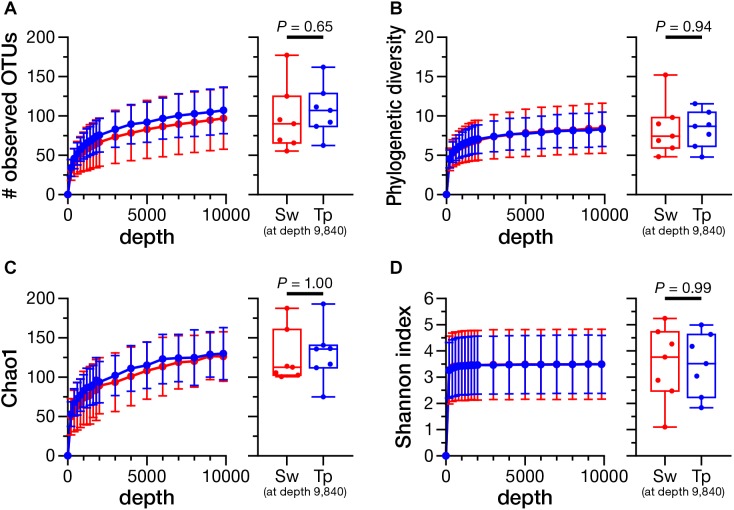
Rarefaction curves and the comparison of alpha diversity indices between the swabbing and tape-stripping methods. A rarefaction analysis on the number of observed operational taxonomic units (OTUs) **(A)**, phylogenetic diversity **(B)**, Chao1 index **(C)**, and Shannon diversity index **(D)**. The analysis was performed up to 9,840 depth. The colors of each plot were assigned as red for the swabbing method and blue for the tape-stripping method. Each index was compared at the rarefaction depth at 9,840. Sw, swabbing method; Tp, tape-stripping method.

The rarefaction analysis at the depth of 9,840 (minimum number of reads) showed no significant differences in the number of observed OTUs (**Figure 4A**; *P* = 0.65), phylogenetic diversity (**Figure 4B**; *P* = 0.94), Chao1 index (**Figure 4C**; *P* = 1.00), and Shannon index (**Figure 4D**; *P* = 0.99) between the swabbing and tape-stripping methods.

### Culture Studies

**FIGURE 5:**
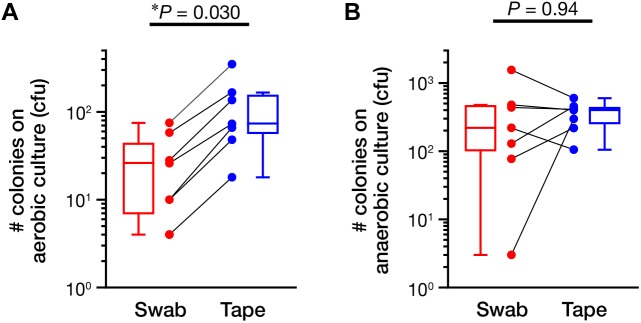
The number of colonies in the culture study. The number of colonies cultured under aerobic **(A)** and anaerobic **(B)** conditions were counted. The data points of the swabbing and tape-stripping methods from the same participant are connected. ^∗^*P* < 0.05. cfu, colony forming unit.

**FIGURE 6:**
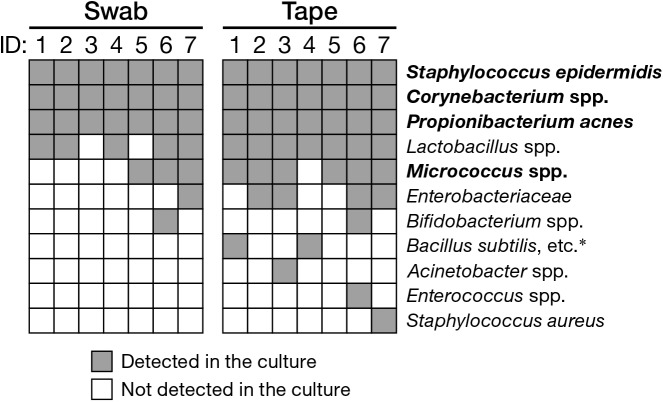
Cultured bacteria using the swabbing and tape-stripping methods confirmed by qualitative PCR. Species-specific primers are as in **Supplementary Table 1**. Bacterial species in bold are skin commensal bacteria. ^∗^*B. anthracis*, *B. thuringiensis*, *B. mycoides*.

Next, we assessed the ability of the swabbing and tape-stripping methods for collection of skin viable bacteria. To collect viable skin bacteria, we employed a traditional culture system with a swab or adhesive tape. The number of cultured colonies collected using the tape-stripping method was significantly higher than that collected using the swabbing method under aerobic conditions (**Figure 5A**, *P* = 0.030); no significant difference was observed under anaerobic conditions (**Figure 5B**, *P* = 0.94). There was a significant correlation in the number of aerobic colonies between the two methods (**Supplementary Figure 6A**; Spearman’s rho = 0.99, *P* < 0.001); however, the correlation was not significant under anaerobic conditions (**Supplementary Figure 6B**; Spearman’s rho = 0.54, *P* = 0.22). **Figure 6** summarizes the cultured skin bacteria obtained using the swabbing and tape-stripping methods. The tape-stripping method collected more abundant cultivable skin bacteria than the swabbing method. Similar results were obtained when different kinds of media (chocolate agar made with trypticase soy agar plus 5% sheep blood, and Columbia agar with 5% sheep blood) were used (**Supplementary Figure 7**).

## Discussion

In this study, we showed that the bacterial composition collected using the tape-stripping method was comparable to that collected using the swabbing method in the NGS analysis, and that the tape-stripping method collected more cultivable bacteria than the swabbing method in the culture study.

We first tested whether the tape-stripping method could reflect the skin microbiome in concordance with the swabbing method, by using NGS analysis. Our results of the NGS analysis showed comparable results between the swabbing and tape-stripping methods in terms of the population of skin microbiome (**Figures 2**–**4**). Intriguingly, the proportion of *Propionibacterium* spp. seemed slightly higher in the tape-stripping group than in the swabbing group (**Figure 2**), although the difference was not significant (**Table 1**). In principle, the swabbing method can mostly capture the outermost, superficial bacteria on the skin (including transient and colonized bacteria), whereas the tape-stripping method can obtain the bacteria inside the stratum corneum by peeling them off. Considering that *Propionibacterium* spp. are aerotolerant anaerobic bacteria, and that the partial seclusion from the outer air by the stratum corneum can create microaerophilic environment (Wilson, 2008), the higher but not significant rate of *Propionibacterium* spp. detected by the tape-stripping method might be a reflection of the difference in the targeting depth of swabbing and tape-stripping methods. Meanwhile, there were several kinds of bacteria (OTUs) that could be detected only by the swabbing method and not by the tape-stripping method (e.g., *Ruminococcus* sp., etc.), and *vice versa* (e.g., *Turicibacter* sp., *Haemophilus* sp., and *Veillonella* sp., etc.) (**Supplementary Tables 4**, **5**). Such differences might account for the bias in the analysis of skin microbiome. However, such “one-sided” bacteria shared very small fractions of the whole population (**Supplementary Tables 4**, **5**, the largest relative abundance was 0.52% in the “swabbing-only” bacteria, and 0.096% in the “tape-stripping-only” bacteria), and some of them had insufficient classification. We consider, therefore, that such minor differences can be almost negligible for most skin microbiome studies. Taken together, it is plausible that the swabbing and tape-stripping methods could be used almost interchangeably for skin microbiome studies in terms of the bacterial composition, unless the very rare or unknown species are targeted.

In the culture study, the tape-stripping method yielded significantly more viable aerobes than the swabbing method (**Figures 5A**, **6**). The tape-stripping method could also stably obtain viable anaerobes as comparable to the swabbing method (**Figures 5B**, **6**). In addition, our preliminary results showed that the repertoires of culturable bacteria collected by using a swab greatly differed among examiners, whereas the tape-stripping method could stably culture skin bacteria (**Supplementary Figure 8**). Such an efficient and stable collection of viable skin bacteria may be explained by the presence or absence of the suspension process rather than by the different collection processes for skin bacteria. In the swabbing method, the collected bacteria should first be suspended in a saline solution, followed by their spreading onto media; whereas in the tape-stripping method, the collected bacteria can be directly cultivated onto media. Another possibility could be, as described above, the difference in the depth where the swabbing and tape-stripping methods are targeting. Meanwhile, we could confirm that the blockage of oxygen by the tape attachment was not the case, as shown by the greater number of aerobes on the tape-stripping medium. In summary, the tape-stripping and swabbing methods can almost equally, with a slight advantage in the number of aerobic bacteria by tape stripping, obtain viable skin bacteria.

We should acknowledge that pore strips have been used for microbiome studies (Fitz-Gibbon et al., 2013; Kang et al., 2015; Barnard et al., 2016; Coughlin et al., 2017). Yet, most of these studies are focusing on the microbiome of comedones (i.e., follicular plugs) on the nose (Fitz-Gibbon et al., 2013; Kang et al., 2015; Barnard et al., 2016), not on that of skin. One study has successfully utilized the pore-strip method for collection of children’s skin bacteria (Coughlin et al., 2017). However, the adhesive strength of a pore strip is much stronger than that of conventional medical adhesive tapes, which requires very careful attention when used for the elderly or young people whose skin is fragile. As our results suggest that the adhesive ability of the medical tape is sufficient to collect skin microbiome, the tape-stripping method with medical tape could be considered as non-invasive, and effective method for skin microbiome studies.

Some limitations regarding the tape-stripping method should be mentioned. First, we used only one kind of adhesive tape; there are several kinds of commercially available adhesive tape that are made of different adhesive glue (e.g., acrylic, silicone, or urethane glue). Further study may be required to find the best adhesive tape (or glue) that can collect as much skin microbiome samples as possible while causing less potential damage to the skin. Second, we could not control the pressure of adhesive tape attachment, thus the same pressure might not have been applied to the skin. However, different examiners who were well-trained in the swabbing and tape-stripping methods but were not instructed about the pressure still detected skin commensal bacteria (e.g., *S*. *epidermidis*, *Corynebacterium* spp., and *P*. *acnes*) more stably by the tape-stripping method than by the swabbing method (**Supplementary Figure 8**). Thus, the attachment pressure may not have much influence on the efficiency of skin bacterial collection. Third, we targeted only a dry, flat skin area (back skin) for the microbiome analysis. The efficiency of collecting skin bacteria by the tape-stripping method may be less when the adhesive tape is attached to wet, oily, and/or undulating skin such as the armpit, scalp, nasal cavity, external auditory canal, or alar crease. Fourth, in the culture experiment, we collected the whole-plate wash, instead of examining each colony. This method could potentially cause bias in the species determination by PCR, because the bacteria that were on the medium but did not grow (i.e., in a viable but non-culturable state) could also be captured by PCR. We have performed the preliminary experiment in which the collected tape from the skin was first attached to the medium, followed by immediate peeling off the tape from the medium without culturing. This procedure can partially mimic the situation where very few numbers of bacteria are remaining on the medium. As a result of PCR of the whole-plate wash, we could not detect any positive signals of bacteria-specific PCR (data not shown). Therefore, we believe that the bacteria that have a very low abundance at the time of harvesting could be excluded by the PCR method in this study. That being said, the comparison of results between culturing and NGS showed a slight inconsistency between the abundance data from NGS and the detection by culturing (**Supplementary Figure 9**). This discrepancy could be explained by bias in the growth of bacteria; even the abundance was very low at first, the culturing could increase the number of bacteria, which leads to the detection by PCR. We should also note that the selection of primers as in **Supplementary Table 1** might lead to detection bias, as the primer sets are not covering all bacterial species, although major skin bacteria such as *S. epidermidis*, *P. acnes*., and *Corynebacterium* spp. are covered. Lastly, we did not compare the bacterial composition between the tape-stripping method versus biopsy or skin-scraping method. The skin biopsy has been considered as being able to offer the most representative skin microbiota, although quite high similarities have been confirmed between the swabbing, skin biopsy, and skin-scraping (Grice et al., 2008).

## Conclusion

In conclusion, the swabbing and tape-stripping methods showed comparable results for skin microbiome analysis, and the tape-stripping method collected more viable bacteria than the swabbing method. The tape-stripping method can be used interchangeably with the swabbing method both for the NGS analysis and for the experiments that require viable skin bacteria, such as antibiotic-susceptibility and virulence tests.

## Data Availability

All raw sequences were deposited in DNA Data Bank of Japan (DDBJ; accession number is DRA006958).

## Author Contributions

KO, JS, and SO conceived the study. KO, SN, and SO performed the experiments. KO, SN, KM, TI, YM, MM, and KS analyzed the data. KO and SO wrote the manuscript. JS and SO acquired the funding. JS and SO supervised the study.

## Conflict of Interest Statement

The authors declare that the research was conducted in the absence of any commercial or financial relationships that could be construed as a potential conflict of interest.
