# Corrigendum: A Comparison of Techniques for Collecting Skin Microbiome Samples: Swabbing Versus Tape-Stripping

**DOI:** 10.3389/fmicb.2018.02812

**Published:** 2018-11-20

**Authors:** Kazuhiro Ogai, Satoshi Nagase, Kanae Mukai, Terumi Iuchi, Yumiko Mori, Miki Matsue, Kayo Sugitani, Junko Sugama, Shigefumi Okamoto

**Affiliations:** ^1^Wellness Promotion Science Center, Institute of Medical, Pharmaceutical and Health Sciences, Kanazawa University, Kanazawa, Japan; ^2^Department of Clinical Laboratory Science, Faculty of Health Sciences, Institute of Medical, Pharmaceutical and Health Sciences, Kanazawa University, Kanazawa, Japan; ^3^Department of Clinical Nursing, Faculty of Health Sciences, Institute of Medical, Pharmaceutical and Health Sciences, Kanazawa University, Kanazawa, Japan; ^4^Advanced Health Care Science Research Unit, Innovative Integrated Bio-Research Core, Institute for Frontier Science Initiative, Kanazawa University, Kanazawa, Japan

**Keywords:** skin microbiome, swabbing, tape stripping, bacterial culture, next generation sequencing

In the original article, there was an error in the sequence of the forward primer used in the real-time PCR.

The forward primer 5′-ACTGAGACACGGYCCA-3′ in the original text should read 5′-ACTGAGAYACGGYCCA-3′. The primer with the corrected sequence was actually used in the study; therefore, the results are not affected.

A correction has been made to Materials and Methods, Real-Time PCR:

To determine the copy number of the 16S rRNA gene in the DNA extracted from the swab or adhesive tape, real-time PCR was performed. The 16S rRNA gene was amplified using universal primer pairs (F: 5′-ACTGAGAYACGGYCCA-3′; R: 5′-CTGCTGGCACGDAGTTAGCC-3′) (Wang and Qian, 2009) and a universal probe (5′-VIC-ACTGCTGCCTCCCGTA-NFQ-MGB-3′) (Gao et al., 2010) with the Thunderbird® Probe qPCR Mix (Toyobo Co., Ltd., Osaka, Japan). A standard curve was drawn from a known amount of the 16S rRNA gene [100, 10, 1, and 0.1 pg of *Propionibacterium acnes* genomes, which are equivalent to 7.23 × 10^4^, 7.23 × 10^3^, 7.23 × 10^2^, and 7.23 × 10^1^ 16S rRNA genes, respectively (Nadkarni et al., 2002; Miura et al., 2010; Stoddard et al., 2015)]. All the reactions were performed with the Mx3005P System (Agilent Technologies, CA, United States). The copy number of 16S rRNA gene was compared for the same size of skin area (4.4 × 4.4-cm square; Supplementary Figure 1, open squares).

The authors apologize for this error and state that this does not change the scientific conclusions of the article in any way. The original article has been updated.

## Conflict of interest statement

The authors declare that the research was conducted in the absence of any commercial or financial relationships that could be construed as a potential conflict of interest.
