# Supplementary material for: A Comparison of Techniques for Collecting Skin Microbiome Samples: Swabbing Versus Tape-Stripping
Source: Front Microbiol. 2018 Oct 2;9:2362. doi: 10.3389/fmicb.2018.02362 (PMC6176111; doi:10.3389/fmicb.2018.02362)
Supplement: Supplementary file 6 [file Data_Sheet_1.PDF]

# *Supplementary Material*

# A Comparison of Techniques for Collecting Skin Microbiome Samples: Swabbing versus Tape-Stripping

**Kazuhiro Ogai[1¶], Satoshi Nagase[2¶], Kanae Mukai[3], Terumi Iuchi[3†], Yumiko Mori[2], Miki Matsue[2], Kayo Sugitani[2], Junko Sugama[3,4], Shigefumi Okamoto[1,2*]**

*** Correspondence:** Shigefumi Okamoto: sokamoto@mhs.mp.kanazawa-u.ac.jp

## 1 Supplementary Figures and Tables

### 1.1 Supplementary Figures

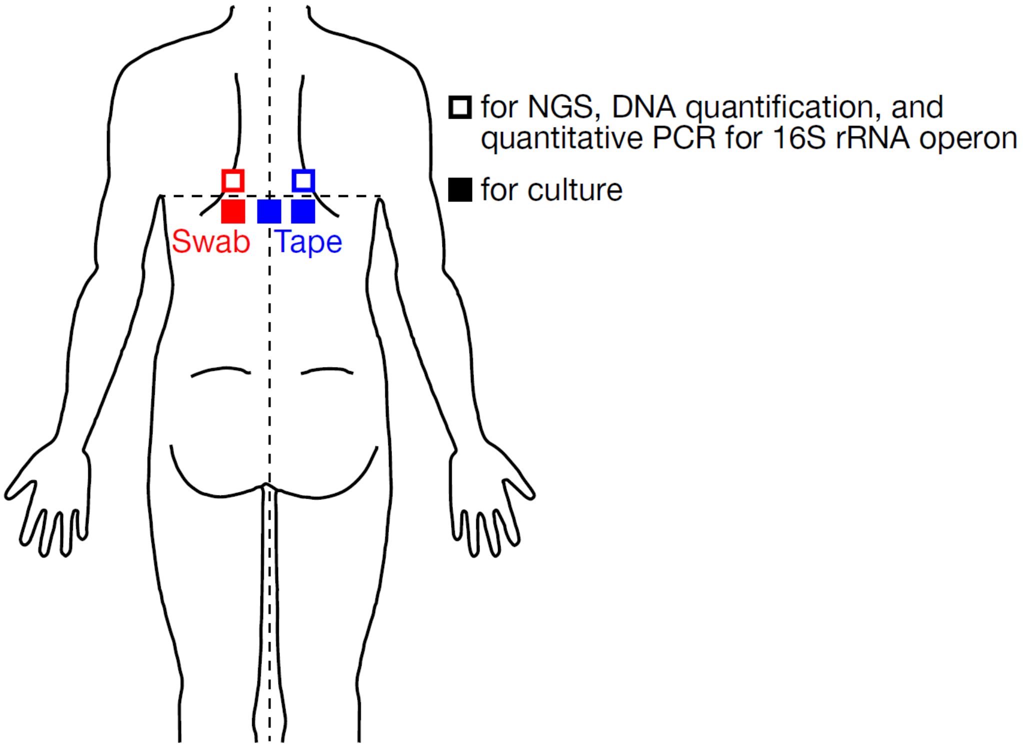


**Supplementary Figure 1.** Position of bacterial collection. The red boxes denote the positions of swabbing and the blue boxes denote those of the tape attachment. The open boxes are for the next-generation sequencing analysis and the closed boxes are for the culture analysis.

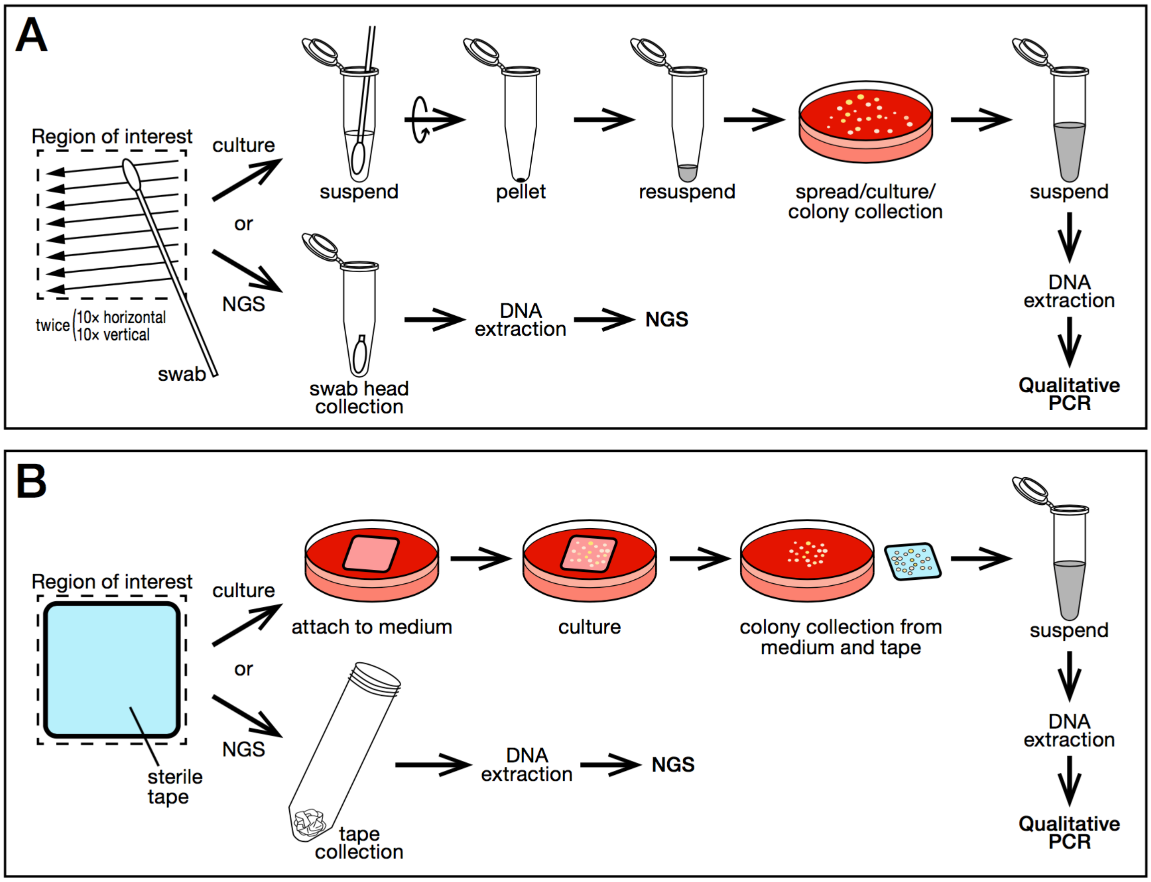


**Supplementary Figure 2.** Flows of the swabbing (**A**) and tape-stripping (**B**) methods are shown. NGS, next-generation sequencing.

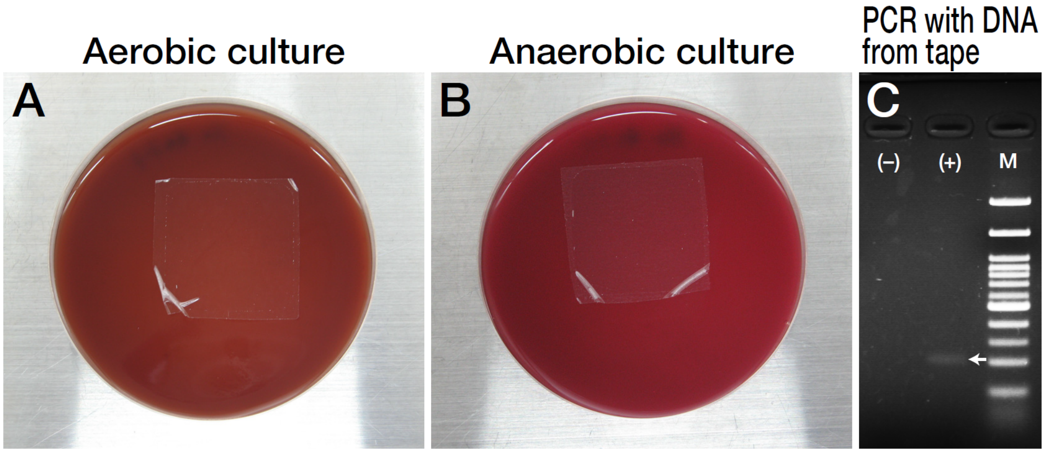


**Supplementary Figure 3.** Sterility and absence of bacterial DNA of the adhesive tape. The UV-irradiated adhesive tapes yielded no bacterial growth on aerobic (**A**) or anaerobic (**B**) culture. (**C**) The adhesive tapes used in this study were confirmed to be free from bacterial DNA by conventional PCR using the 16S rRNA gene universal primers. The arrow indicates the amplified 16S rRNA gene fragment. (−), tape only; (+), tape attached to the skin; M, 100 bp ladder marker.

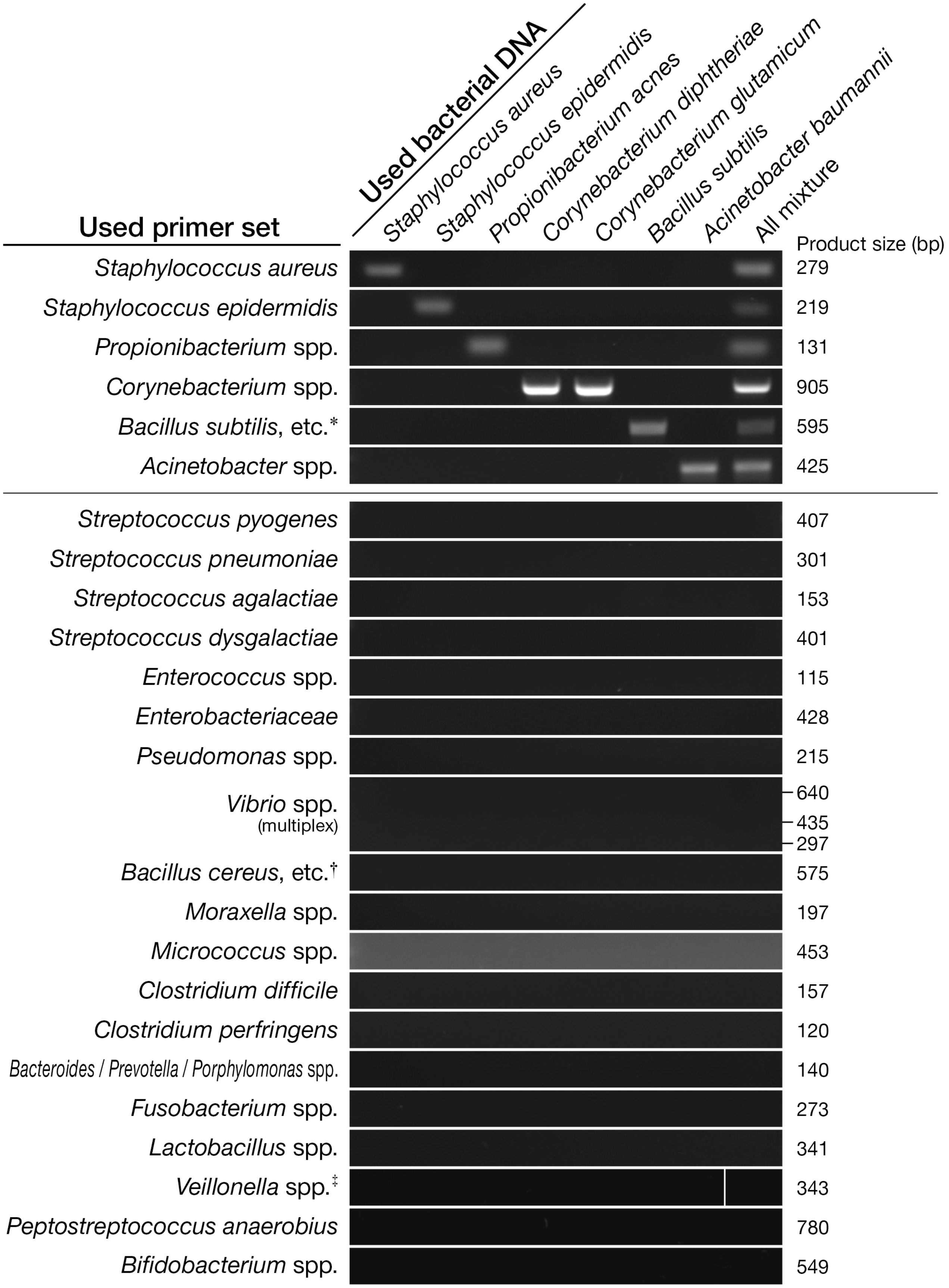
Used bacterial DNA
Staphylococcus aureus
Staphylococcus epidermidis
Propionibacterium acnes
Corynebacterium diphtheriae
Corynebacterium glutamicum
Bacillus subtilis
Acinetobacter baumannii
All mixture
Used primer set
Product size (bp)
Staphylococcus aureus 279
Staphylococcus epidermidis 219
Propionibacterium spp. 131
Corynebacterium spp. 905
Bacillus subtilis, etc.* 595
Acinetobacter spp. 425
Streptococcus pyogenes 407
Streptococcus pneumoniae 301
Streptococcus agalactiae 153
Streptococcus dysgalactiae 401
Enterococcus spp. 115
Enterobacteriaceae 428
Pseudomonas spp. 215
Vibrio spp. (multiplex) 640 435 297
Bacillus cereus, etc.† 575
Moraxella spp. 197
Micrococcus spp. 453
Clostridium difficile 157
Clostridium perfringens 120
Bacteroides / Prevotella / Porphylomonas spp. 140
Fusobacterium spp. 273
Lactobacillus spp. 341
Veillonella spp.‡ 343
Peptostreptococcus anaerobius 780
Bifidobacterium spp. 549

**Supplementary Figure 4.** Specificity of each primer set. Each primer set was reacted with several kinds of bacterial DNA (*Staphylococcus aureus* ATCC 6538P, *S. epidermidis* ATCC 14990, clinically-isolated *Propionibacterium acnes*, clinically-isolated *Corynebacterium diphtheriae*, *C. glutamicum* ATCC 13032, *Bacillus subtilis* ATCC 6051, and clinically-isolated *Acinetobacter baumannii*) as well as the mixture of all bacterial DNA. PCR amplification was achieved only when the primer set and bacterial DNA matched. [*]*B. anthracis*, *B. thuringiensis*, *B. mycoides*. [†]*B. licheniformis*, *B. amyloliquefaciens*, *B. pumilus*, *B. atrophaeus*. [‡]The last lane was electrophoresed in another lane and then combined.

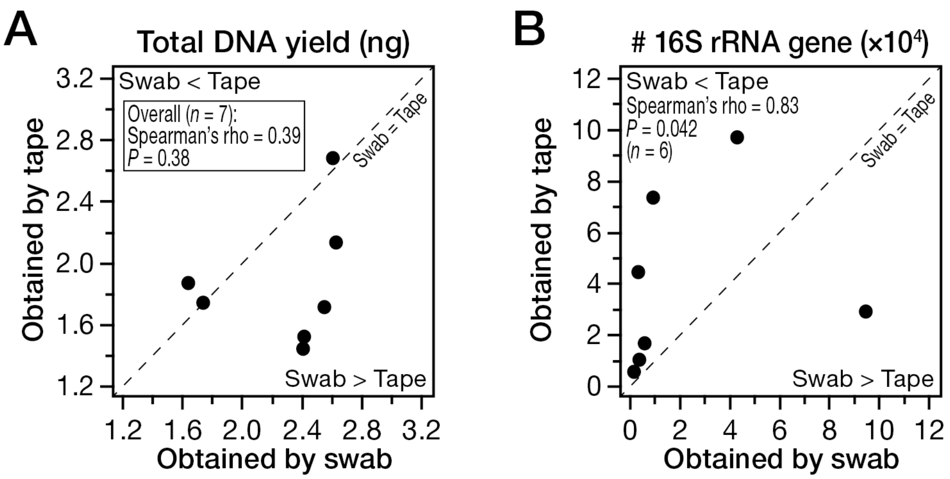


**Supplementary Figure 5.** Correlations in the total DNA yield (**A**) and the number of 16S rRNA gene (**B**) between swabbing and the tape-stripping method.

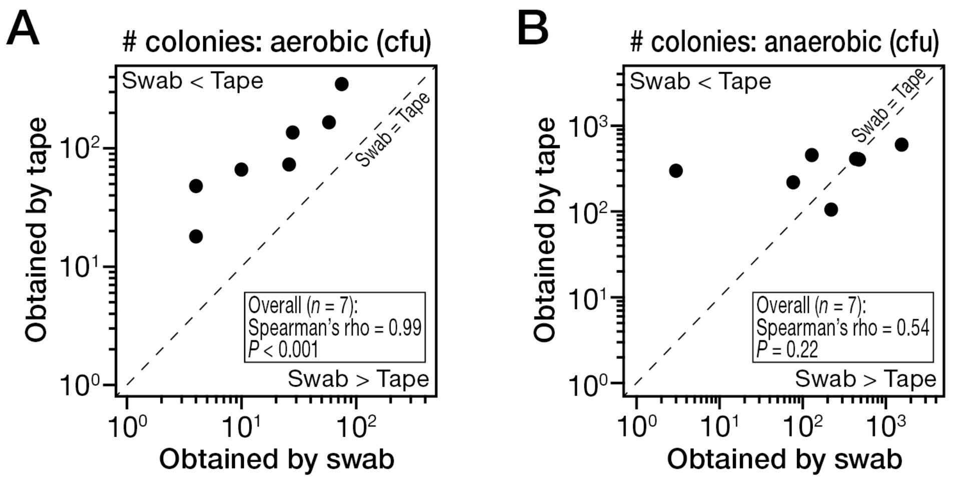


**Supplementary Figure 6.** Correlations in the number of colonies under aerobic conditions (**A**) and anaerobic conditions (**B**).

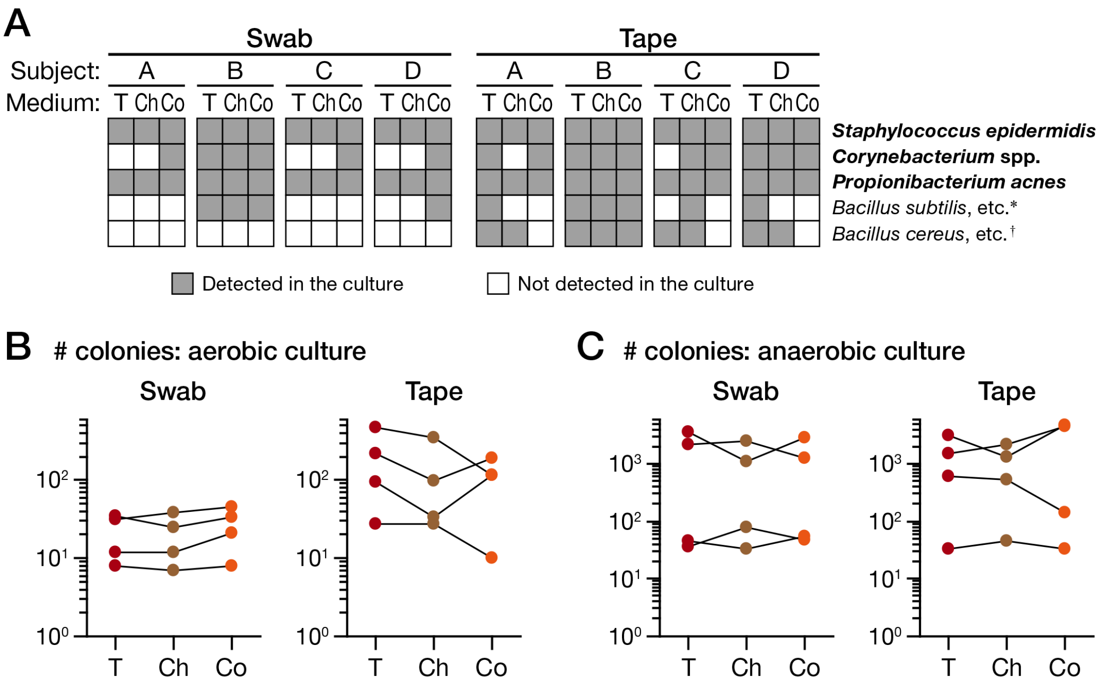


**Supplementary Figure 7.** Differences in the detected (cultured) bacteria (**A**) and numbers of aerobic (**B**) and anaerobic (**C**) colonies by different media. Three different kinds of unselective media were used: T, trypticase soy agar with 5% sterile defibrinated sheep blood; Ch, chocolate agar; Co, Columbia agar with 5% sheep blood. Bacterial species in bold are skin commensal bacteria. **B. anthracis*, *B. thuringiensis*, *B. mycoides*. *†B. licheniformis*, *B. amyloliquefaciens*, *B. pumilus*, *B. atrophaeus*.

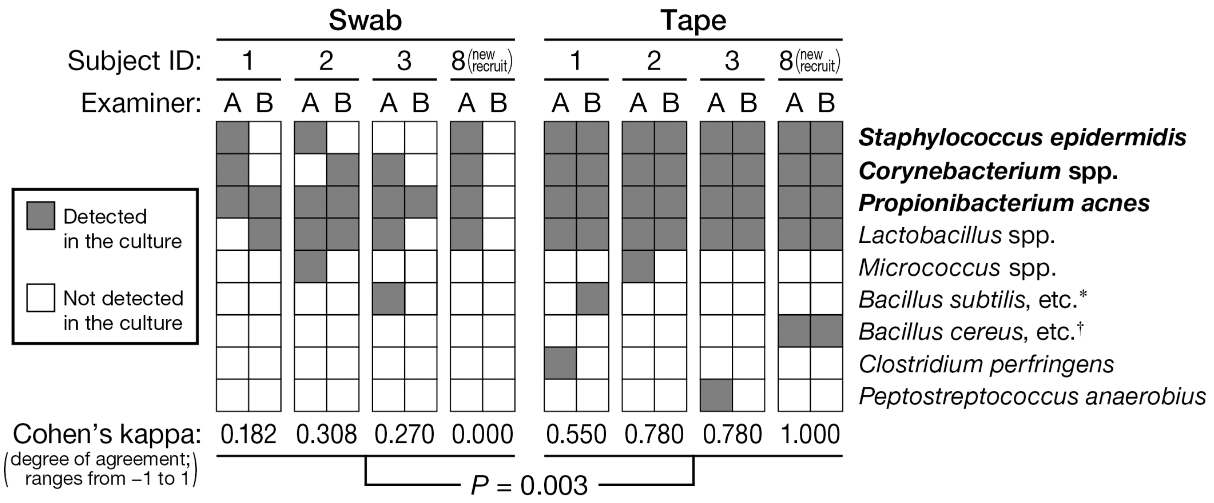


**Supplementary Figure 8.** Comparison of cultured bacteria between two examiners with the different methods of skin microbiota collection. Cohen's kappa coefficients [ranges from −1 (complete disagreement) to 1 (complete agreement)] were calculated and compared using Welch's *t*-test. Bacterial species in bold are skin commensal bacteria. **B. anthracis*, *B. thuringiensis*, *B. mycoides*. *†B. licheniformis*, *B. amyloliquefaciens*, *B. pumilus*, *B. atrophaeus*.

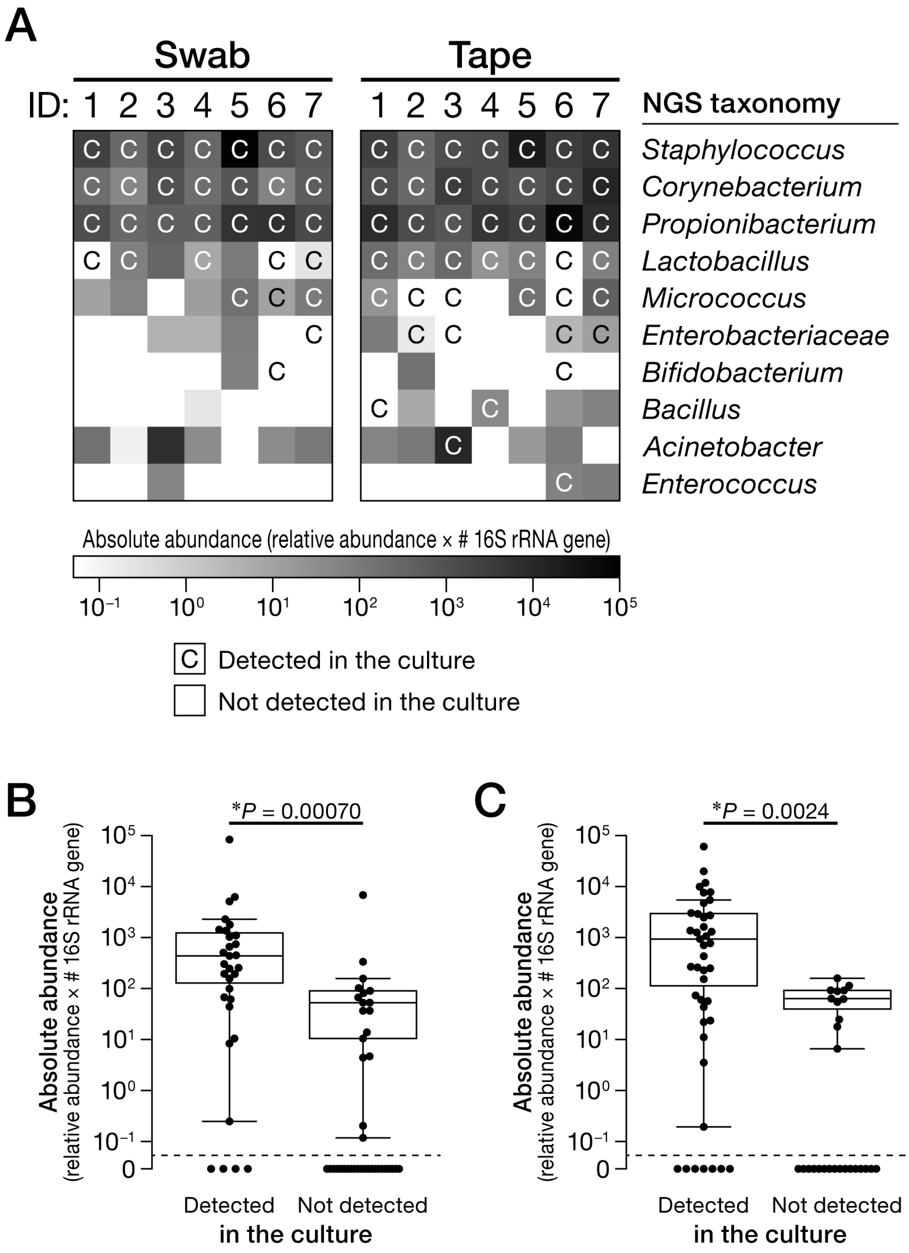


**Supplementary Figure 9.** Comparison of the results between cultured bacteria and NGS. (**A**) Heatmaps of the absolute abundance (relative abundance × the number of 16S rRNA gene) with the results of cultured bacteria (the letter "C" represents the positive culture). The absolute abundance was compared between the cultured and not-cultured bacteria in swabbing (**B**) and the tape-stripping method (**C**). Generally, higher abundance in the NGS analysis was related to the positive culture. $^*P < 0.01$; Mann–Whitney $U$ test.

## 1.2 Supplementary Tables

**Supplementary Table 1.** Species-specific primer sets.

**Supplementary Table 2.** Primers for 2nd PCR of next generation sequencing.

**Supplementary Table 3.** Read numbers in each sequence processing.

**Supplementary Table 4.** Bacteria detected by the swabbing method but not by the tape-stripping method.

**Supplementary Table 5.** Bacteria detected by the tape-stripping method but not by the swabbing method.
